# CD146, a novel target of CD44-signaling, suppresses breast tumor cell invasion

**DOI:** 10.1186/s12964-017-0200-3

**Published:** 2017-11-09

**Authors:** Allal Ouhtit, Mohammed E. Abdraboh, Andrew D. Hollenbach, Hatem Zayed, Madhwa H. G. Raj

**Affiliations:** 1 Department of Biological and Environmental Sciences, College of Arts and Sciences, Qatar University, Doha, Qatar; 2 Department of Zoology, Faculty of Science, Mansoura University, Mansoura, Egypt; 3 Department of Genetics, Louisiana State University, Health Sciences Center, New Orleans, USA; 4 Department of Biomedical Sciences, College of Health and Sciences, Qatar University, Doha, Qatar; 5 Department of Obstetrics and Gynecology, Louisiana State University, Health Sciences Center, New Orleans, USA

**Keywords:** CD146, CD44, Breast cancer, Metastasis, MMPs

## Abstract

**Background:**

We have previously validated three novel CD44-downstream positively regulated transcriptional targets, including Cortactin, Survivin and TGF-β2, and further characterized the players underlying their separate signaling pathways. In the present study, we identified CD146 as a potential novel target, negatively regulated by CD44. While the exact function of CD146 in breast cancer (BC) is not completely understood, substantial evidence from our work and others support the hypothesis that CD146 is a suppressor of breast tumor progression.

**Methods:**

Therefore, using molecular and pharmacological approaches both in vitro and in breast tissues of human samples, the present study validated CD146 as a novel target of CD44-signaling suppressed during BC progression.

**Results:**

Our results revealed that CD44 activation could cause a substantial decrease of CD146 expression with an equally notable converse effect upon CD44-siRNA inhibition. More interestingly, activation of CD44 decreased cellular CD146 and increased soluble CD146 through CD44-dependent activation of MMP.

**Conclusion:**

Here, we provide a possible mechanism by which CD146 suppresses BC progression as a target of CD44-downstream signaling, regulating neovascularization and cancer cell motility.

## Background

Cell adhesion molecules (CAMs) are glycoproteins responsible for promoting cell-cell adhesion and cell-extracellular matrix interactions [1]. Most cancerous cells exhibit loss of cell adhesion capability due to alteration in CAMs, which mediate the movement of cancer cells from their tissue of origin to the secondary sites of metastasis [2]. Our research efforts have been focused on understanding the mechanisms by which CD44 promotes breast tumor invasion [3–5]. CD44 is a member of the immunoglobulin-like family, known to promote BC metastasis through binding of CD44 to its main ligand hyaluronan (HA), or to other ligands, such as osteopontin, fibronectin, collagen IV, and laminin [6]. The binding of HA to CD44 initiates a series of cell signaling events through the activation of CD44 C-terminal bound proteins such as Ankyrin, merlin, and ERM (Ezrin/Radixin/Moesin). The CD44 cytoplasmic tail is directly associated with actin filaments in a process mediated by ERM proteins [7]. In order to identify the transcriptional targets of CD44-HA interactions mediating breast tumor invasion, we initially employed two molecular approaches: i) subtractive hybridization and microarray analyses, using the two BC cell lines, MDA-MB-231 (highly metastatic cells expressing high levels of CD44) and MCF-7 (weakly invasive BC cells without any noticeable CD44 expression); and ii) The MCF-7 founder cell lines were used to establish the CD44-tet Off inducible system. RNA samples from both CD44-induced and non-induced cells were compared using microarray analysis. Microarray analysis revealed dozens of potential CD44-downstreatm transcriptional targets. Among these targets, we have already validated cortactin [3], survivin [4] and TGF-β2 [5] as CD44-positively regulated targets, and further characterized their respective underlying molecular players for each signaling pathway mediating CD44-dependent BC cell survival and motility (CD44/Cortactin, CD44/Survivin and CD44/ TGF-β2). However, substractive hybridization experiments led to the identification of the CAM, CD146 as a potential downstream target, negatively regulated by CD44-signaling (unpublished data). Therefore, the present study focused on the structural and functional validation of CD146, and provided evidence that CD146 is a downstream target of CD44, suppressed during BC cell invasion.

CD146, also known as MCAM or Mel-CAM and a member of the immunoglobulin-like CAM family, is activated through a dimerization of its ligand, leading to a cascade of signal transduction events (reviewed in [2]). CD146, a known marker of endothelial cells [8], is commonly used marker for the prognosis of patients with metastatic cancer [9]. Soluble CD146 (sCD146) is known to play a key role in angiogenesis. In fact, Lin et al. (2007), showed that suppression of CD146 expression in epithelial progenitor cells (EPC) led to a significant reduction in recombinant CD146-stimulated angiogenesis, probably due to decreased expression of pro-angiogenic factors [10]. In another study, CD146 promoted angiogenesis via induction of pro-angiogenic factors, such as vascular endothelial growth factor (VEGF), urokinase plasminogen activator (uPA), endothelial nitric oxide synthase (eNOS) and matrix metalloproteinase 2 (MMP2) [11]. On the other hand, Kebir et al. (2010) demonstrated that the short isoform of CD146 promoted angiogenesis via stimulating adhesion of circulating progenitor cells (CPC) to activated human vascular endothelial cells [12].

CD146 was first identified as a viable biomarker for melanoma [13, 14], due to its high expression in 80% of melanomas, particularly in advanced primary and metastatic tumors [15, 16]. More interestingly, while CD146 was detected in highly metastatic prostate cancer (PC) cell lines such as PC3 and DU145, it was not detected in non-metastatic PC cell line such as LNCaP and normal prostate cells [17, 18]. High levels of CD146 expression were also associated with progression of prostate adenocarcinoma in a mouse TRAMP model [19].

While CD146 is a promoter of melanoma and PC, its role in BC remains nascent and controversial. In fact, while some reports support the role of CD146 as a promoter of BC cell growth and metastasis [19–21], others, including our own work, have demonstrated the role of CD146 as an inhibitor of BC cell growth and invasion [2, 22, 23]. The rationale of this study is based on the following observations: i) substantial evidence from the literature indicate that CD146 can suppress BC growth and invasion [2, 22, 23]; and ii) Our gene expression profiling data revealed a six-fold down-regulation of CD146 upon HA-mediated activation of CD44 (data not shown). Therefore, based on these observations, we hypothesized that CD44 down-regulates CD146 expression during breast tumor progression. The aim of this study was to validate CD146 as a negative transcriptional target of CD44-HA downstream signaling mediating breast tumor cell invasion. In addition to better understand the relationship between CD44 and CD146, this investigation has the potential to provide mechanistic evidence of the role of CD146 as a tumor suppressor of BC via CD44-HA interactions.

## Methods

### Cell culture

The previously described MCF7-B5 cell line, a primary BC cell line stably transfected with a tetracycline-regulated CD44 vector (tetracycline off) [3], was stably co-transfected with a vector that constitutively expresses CD146 to obtain MCF7-B5-CD146 also called B4 cells. The MCF7-B5 and MCF7-B5-CD146 cells were cultured in Dulbecco’s Modified Eagle’s Medium (DMEM) containing 10% (*v*/v) fetal bovine serum (FBS), 2 mmol/L _L_-glutamine, 1 mmol/L sodium pyruvate (Gibco, Gaithersburg, MD), 2.5 μg/ml doxycyline (a tetracycline analog with greater chemical stability), 100 μg/ml G418 (Roche Diagnostics Ltd. (GmBH), Lewes UK), and 1 μg/ml puromycin (Invivogene, CA). To induce the expression of CD44, the cells were cultured in the same media in the absence of doxycyline but with the addition of 50 μg/ml Zeocin (Invivogene, CA), selecting agent of CD146.

### Western blot analysis

Protein lystaes (40 μg) were prepared as previously described (Ouhtit et al., 2007) and boiled for 5 min in an equal volume of reducing buffer (5 mmol/L of Tris/HCl (pH 7.4), 4% (*w*/*v*) sodium dodecyl sulfate, 20% (*v*/v) glycerol, 10% (v/v) mercaptoethanol, 0.2% (w/v) bromophenol blue), resolved on 12% polyacrylamide gels and electroblotted onto nitrocellulose membranes. Membranes were probed with mouse monoclonal anti-CD44 (1:1000 dilution; R&D Biosystems, MN), a mouse monoclonal anti-CD146 (1:500 dilution; Novocastra HD, Leica Biosystems, UK), and goat anti-actin antibodies (1:500 dilution; Santa Cruz Biotechnology, CA), followed by incubation with donkey anti-mouse (1:2000 dilution; Santa Cruz Biotechnology, CA) and donkey anti-goat IgG-HRP (1:2000 dilution; Santa Cruz Biotechnology, CA) secondary antibodies. The presence of the protein was detected using the West Femto Supersignal chemiluminescence kit (Thermo Scientific, IL).

### RNAi-mediated depletion of CD44 and CD146

Oligonucleotides specific for human CD44, human CD146, along with a Silencer® Negative Control #1 siRNA were synthesized commercially (Ambion, TX) for the siRNA knockdown of CD44. The sequences used to inhibit CD44 are, 5′-GGAAAUGGUGCAUUUGGUGTT-3′ (sense) and 5′-TTCCUUUACCACGUAAACCAC-3′ (antisense), and for CD146 are, 5′-GGCACAGCUGGUUAAAGAATT -3′ (sense) and 5′-UUCUUUAACCAGCUGUGCCTT-3′ (antisense).

Cells were seeded in 6-well dishes and grown to approximately 50% confluency, washed twice with sterile PBS and then incubated with a transfection cocktail containing OPTI-MEM, Lipofectamin-2000 (Invitrogen, CA) and the siRNA or scrambled oligonucleotides at a final concentration of 50 nM for CD44 and CD146 at 37 °C for 6–7 h in DMEM culture media without doxycyline supplemented with 20% bovine serum albumin (BSA). After incubation, the media was replaced with 10% (*v*/v) FBS-enriched growth media and subsequently incubated overnight at 37 °C. Transfected cells were re-transfected (24 h after the first transfection), as described to ensure CD44 or CD146 inhibition. Total cell extracts were prepared within 48 h after the second transfection and protein expression was confirmed by Western blot analysis, as described above.

### Immunofluorescence

The cells were treated with CD44 siRNA as described above and fixed with 2% paraformaldehyde in PBS. Cells were washed twice with PBS, blocked with 1% BSA/PBS and incubated overnight at 4 °C with rabbit anti-CD44 (1:30 dilution; Santa Cruz Biotechnology, CA) and mouse anti-CD146 (1:30 dilution; Novocastra HD, Leica Biosystems, UK) primary antibodies, prepared in 1% BSA/PBS. Cells were washed and incubated with Alexa Fluor 488 or 546 (Invitrogen, CA) for 1 h at room temperature. Slides were mounted in Vectashield (Vector Laboratories, CA) and the images were captured using a Laser Scanning Confocal Microscope (Bio-Rad).

### Cell invasion assay

MCF7-B5-CD146 cells were cultured in the presence of dox and Zeocin in the absence of HA (100 μg/ml) for a period of 24 h in order to inhibit CD44 expression (CD44-,CD146+). Cells were then transfected with 50 nM of human CD146 specific siRNA as described above to inhibit CD146 expression and 24 h after siRNA treatment; the cells were washed twice with sterile PBS and harvested by trypsinization. The harvested cells (5.0 × 10^4^) were then re-suspended in DMEM supplemented with 0.5% BSA and plated in Millicell culture inserts (8 μm pore size, Millipore, MA), which were previously coated with a thin layer of 200 μg/ml of Matrigel™ (BD Biosciences, MA). The inserts containing the cells were placed into a tissue culture dish (lower chamber) containing the “attracting medium”, consisting of MCF7-B5-CD146 special medium as stated above. Cells were incubated for 22 h at 37 °C and the Millicell culture insert was removed and the upper surface of the insert was wiped immediately with a cotton swab in order to remove non-invasive cells. The cell culture inserts were dried under laminar flow hood for 4 h, and the cells that had invaded and were present at the bottom of the filter were stained using the Diff-Quick staining kit, according to the manufacturers’ protocol (Dade Behring Inc.). The stained cells were counted under a phase-contrast microscope, equipped with ocular grids. Data was presented as Mean ± Standard deviation of at least triplicates from three experiments and statistically analyzed by SPSS program using Student’s *t*-test. The difference is considered statistically significant when *p* < 0.05.

### Immunohistochemistry

Paraffin blocks including normal and tumor breast tissue were obtained from the archives of the Department of Pathology at Louisiana State University Health Sciences Center, New Orleans. Immunohistochemical assays were performed by examining adjacent sections for both CD44 and CD146 expression using mouse anti-human CD44 antibody (1:100 dilution, R&D, CA) and polyclonal mouse anti-CD146 antibody (1:25 dilution, Novocastra) after antigen retrieval carried out by boiling the samples in 500 ml of 9 mM citrate buffer (pH 6) (Invitrogen, Carlsbad, CA) for 25 min. Primary antibodies were applied overnight at 4°C. Biotinylated secondary antibody of vector labs VECTASTAIN ABC systems universal kit and DAB substrate were applied according to the manufacturers specifications (Vector labs, CA). For the intensity of immunostaining, we adopted a simple comparison of the intensity of immunostaining using 1+ for low expression, 2+ for intermediate expression and 3+ for high expression.

### Zymography

MCF7-B5-CD146 cells were cultured for 24 h in (HA-, Tet+) supplemented media to induce CD146 expression at the cell surface. The culture media was then replaced with a (HA+, Tet-) supplemented media in order to induce CD44 expression. Following culturing, the culture media was collected at 0, 24 and 48 h concentrated using Millipore minicon concentrator columns (Millipore, MA) and was frozen at -80 °C till use. Sample aliquots of 30 μg were separated using casein zymography pre-casted gels according to the manufacturer’s protocol (Invitrogen, CA).

### Statistical analysis

Differences were assessed for statistical significance by SPSS program using Student’s *t*-test two-tailed. Data was presented as Mean ± Standard deviation of at least triplicates from three experiments. The difference is considered statistically significant when *p* < 0.001 (*).

## Results

### The inverse relationship between cellular CD44 and CD146 in MCF7-B5 cells

**Fig. 1:**
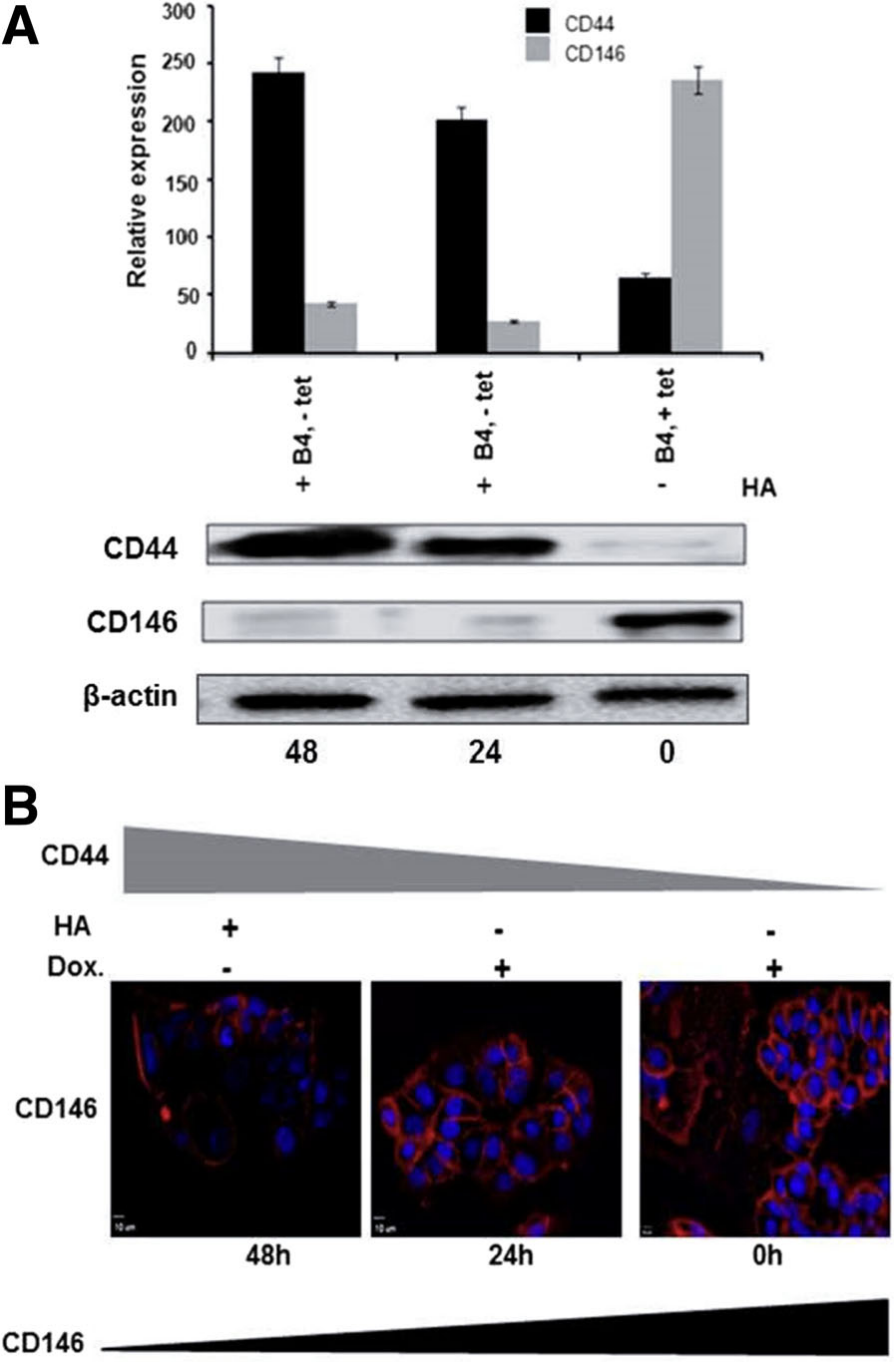
Western blot and immunocytochemical analyses demonstrating the inverse relationship between CD44 and CD146 expression. **a** MCF7-B5-CD146 cells (B4 cells) in the absence (−Dox) or presence (+Dox) of induced CD44 expression were activated with the CD44 ligand, Hyaluronan (HA). Total cell extracts were made and the presence of CD44, CD146, or beta-actin (loading control) at 0, 24, 48 h was determined by Western blot analysis. Shown are the cropped blot images representing indicated proteins, and all the gels from three separate experiments have been run under the same experimental conditions. **b** Immuno-fluoresence staining of CD146 in MCF7-B5-CD146 cells, indicates the upregulation of CD146 expression at the cell surface in the absence of CD44 (Dox +, HA-). Data was presented as Mean ± Standard deviation of at least triplicates from three separate experiments. The difference is considered statistically significant (student’s two-tailed t-test, *P* < 0.001)

The majority of previous studies related to CD146 in BC focused generally on the detection of its expression in circulating endothelial cells, present in the blood of BC patients [24, 25]. In contrast, only few reports examined the expression of CD146 in BC [21, 26], and yet however, significant discrepancies exist. In fact, while some reports concluded that CD146 promotes BC cell growth and metastasis [20, 21], other studies showed that CD146 inhibits BC progression [2, 22, 23]. To address this discrepancy, we have initially employed two molecular approaches: subtractive hybridization and microarray analyses using the two BC cell lines, MDA-MB-231 (high CD44 expression) and MCF-7 (no CD44 expression) tetracycline (Tet)-off CD44-inducible cells. Our results revealed a six-fold down-regulation of CD146 when CD44 was activated by HA (data not shown). Molecular and functional approaches were employed in the present study to validate CD146 as a negative transcriptional target of CD44-HA signaling bot at structural and functional levels. First, we examined the expression of CD44 and its potential target CD146 in BC cells. In order to investigate the relationship between CD44 and CD146, MCF7-B5 cells, which allow the inducible expression of CD44, were stably co-transfected with a construct allowing the constitutive expression of CD146 (MCF7-B5-CD146 [3–5], also named B4 clone). The expression of CD44 was induced by the removal of doxycycline (dox) and activated by the addition of HA. The expression levels of CD44 and CD146 were then determined by Western blot analysis (Fig. 1a). A significant induction of CD44 expression was observed upon the removal of dox (Fig. 1a). Interestingly, a distinct inverse relationship between CD44 and CD146 expression was observed, with a significant reduction in CD146 levels upon induction and activation of CD44. In addition, induction of CD44 decreased the levels of CD146 regardless of the presence or absence of HA (data not shown). This results suggest that CD44 downregulates CD146 through other known CD44-ligands or via other unknown mechanism. Similarly, immuno-fluorescence staining of CD146 in MCF7-B5-CD146 cells, showed an upregulation of CD146 expression at the cell surface in the absence of CD44 (Fig. 1b).

**Fig. 2:**
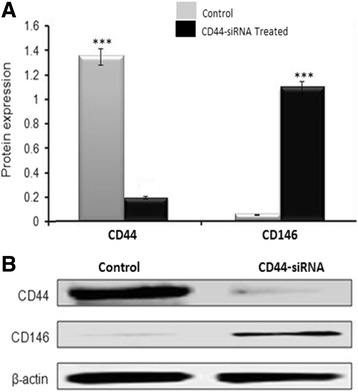
Structural validation of the relationship between CD44 and CD146. Immunoblot data illustrating the inhibition of CD44 expression in B4 cells using 50 nM of human specific CD44-siRNA. The CD44 inhibition resulted in a marked rescue of CD146 expression. Shown are the cropped blot images representing indicated proteins, and all the gels from three separate experiments have been run under the same experimental conditions. Densitometry was carried out to measure protein expression levels for both CD44 and CD146 (both control and treated sample are shown in the histogram). The values of protein expression levels shown in the histogram are representative of the average calculated from triplicate experiments. The difference is considered statistically significant (student’s two-tailed t-test, *P* < 0.001)

To further validate these results, an alternative approach in which CD44 expression was inhibited by human specific CD44 siRNA was employed. Treatment of the cells with CD44-specific siRNA effectively inhibited the expression of CD44 (Fig. 2). Consistent with the results shown in Fig. 1b, we observed a significant increase in the expression of cellular CD146 upon siRNA specific inhibition of CD44 (Fig. 2).

### Expression of CD146 and CD44 in breast tumor tissues

**Fig. 3:**
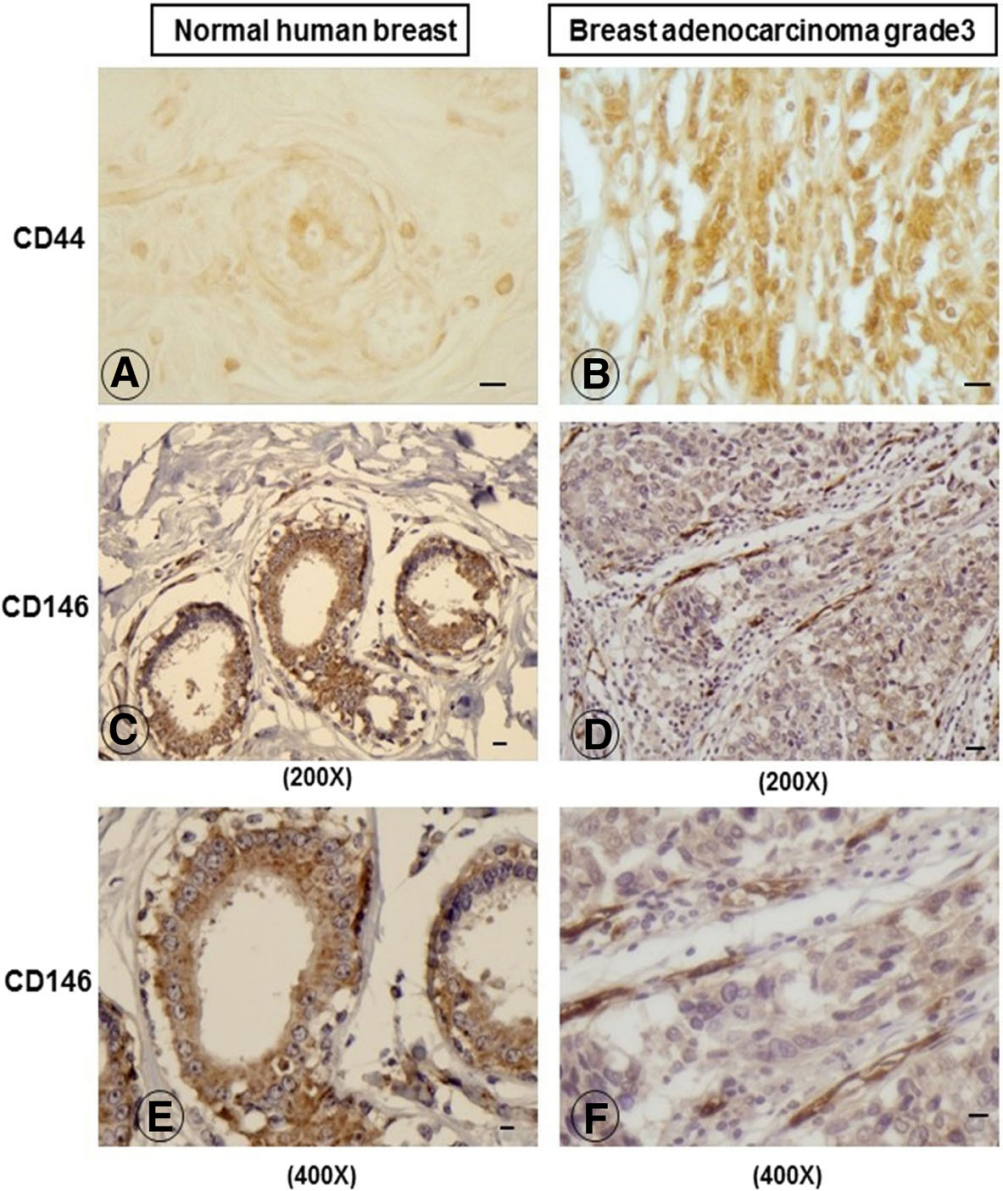
Protein expression levels of CD44 and CD146 in human normal and breast tumor tissues. Immunohistochemical analyses of CD44 expression in normal human breast tissue **a** 200X and in grade 3 breast adenocarcinoma tumor tissues; **b** 200X. Expression patterns of CD146 in normal breast tissue; **c** 200X and in grade 3 breast adenocarcinoma tumor tissues **d** 200X; **e** 400X. grade 3 breast adenocarcinoma tumor tissues and; **f** 400X

Next, we examined both normal and tumor breast tissue samples from four patients with highly aggressive grade 3 breast adenocarcinoma, for the expression levels of CD44 and CD146, by immunohistochemical analysis. The neighboring normal human breast tissue from the same patient was used as a control. We observed low CD44 staining (1+) in the normal human breast tissue with a strong staining (3+) for CD146 (Fig. 3a, c, and e, and Table 1). In contrast, the breast tumor sample contained high levels (3+) of CD44 expression with the concomitant low (1+) CD146 expression levels (Fig. 3b, d, and f, and Table 1), and these findings were consistent with our Western blotting results (Fig. 1). Interestingly, the low (1+) CD146 staining present in the breast tumor tissue was specifically localized in blood vessel endothelial cells, consistent with its known role as a marker for endothelial cells [8].

**Table 1:** Inverse relationship between the protein expression levels of CD44 and CD146 in human normal and breast tumor tissues

	Intensity of immunostaing in normal breast tissue		Intensity of immunostaing in the breast adenocarcinomas grade 3 tumors	
Patient number	CD44	CD146	CD44	CD146
1	+	+++	+++	+
2	+	++	+++	+
3	+	+++	+++	+
4	+	+++	+++	+

A simple comparison of the intensity of immunostaining was adopted using 1+ for low expression, 2+ for intermediate expression and 3+ for high expression, and the data was summarized in a table

### Cellular localization of CD44 and CD146

**Fig. 4:**
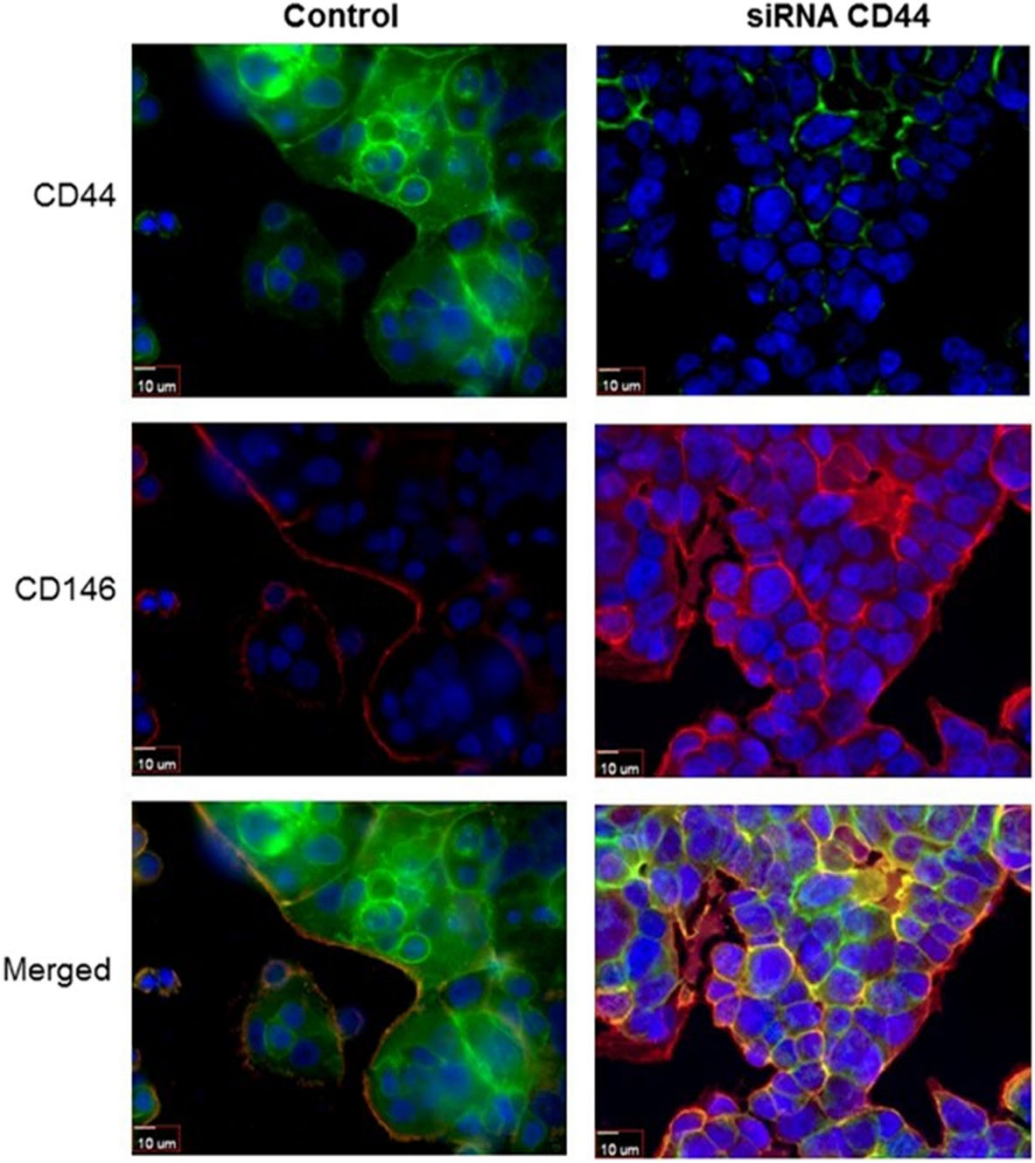
Structural validation of the inverse relationship between CD44 and CD146. Double Immuno-cytochemical staining of CD44 and CD146 in scrambled siRNA (control) and CD44 siRNA treated MCF7-B5-CD146 cells. The CD44 specific inhibition by siRNA resulted in a marked rescue of CD146 expression

To further examine the effect of CD44 on CD146 expression, we examined the cellular localization of both these proteins by immunofluorescence. We observed a perceptible reduction of CD44 expression on the cell surface of the MCF7-B5-CD146 cells upon treatment with CD44-specific siRNA; a phenomenon not observed using the non-specific control siRNA construct (Fig. 4). Consistent with our Western blot results, downregulation of CD44 expression was accompanied by visible increase in the cell surface expression of CD146 (Fig. 4). Interestingly, low expression (1+) of the cell surface CD146 was observed in the presence of CD44. However, this CD146 expression was restricted to cellular surfaces adjacent to “empty spaces” and was not present in regions of cell-cell contact (Fig. 4, control). Further, upon the decrease of CD44 expression, CD146 was subsequently expressed on all cellular surfaces, including those mediating cell-cell interactions (Fig. 4, siRNA CD44). Taken together, these results demonstrate an inverse relationship between the expression of CD44 and CD146 in MCF7 BC cells.

### Cell surface vs soluble CD146

**Fig. 5:**
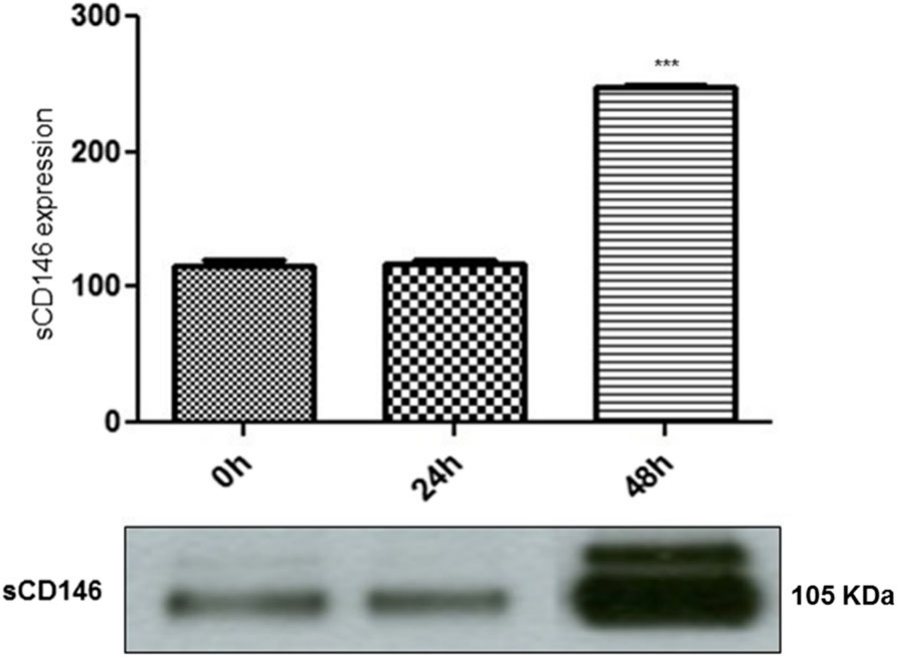
Western blot analysis demonstrating the increase of sCD146 level upon CD44 up-regulation. The expression of CD44 in (MCF7-B5-CD146) was stimulated (HA+, Dox-), the culture medium was collected at 0, 24 and 48 h. The collected media were concentrated and tested for the level of CD146 ectodomain (sCD146). Shown is the cropped blot image representing indicated protein (sCD146; ~105 kDa), and the gel has been run, for the three samples, under the same experimental conditions. The difference is considered statistically significant (student’s two-tailed t-test, ***P < 0.001)

The inverse relationship between CD44 and CD146 cellular levels, illustrated in the data described above prompted us to further examine the mechanism by which CD44 controls CD146 at cellular levels. In order to test this mechanism we collected culture media of MCF7-B5-CD146 cells after induction of CD44 expression (−Dox, +HA). One big band correspondingThe protein levels of both CD44 and CD146 were determined using Western blot analysis. Our data showed significant increase of sCD146 levels in the culture media after 48 h of CD44 induction (Fig. 5). These results suggest that CD44 might down-regulate CD146 at cellular levels, perhaps through CD44-dependant detachment of CD146 from the cell surface into the culture medium.

### Expression of CD44 promotes the MMP-dependent release of CD146 from the cell surface

**Fig. 6:**
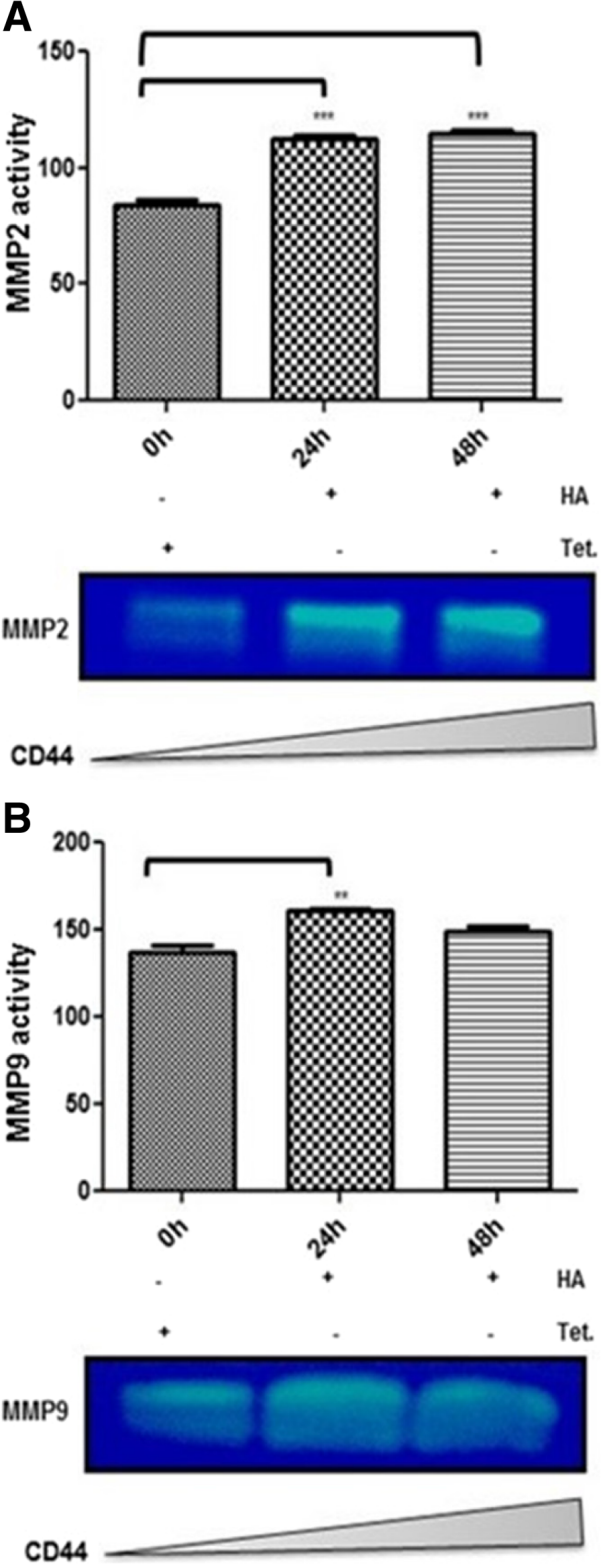
Zymography approach showing the increase in MMPs activity upon CD44 up-regulation. **a** MCF7-B5-CD146 cells were cultured in the absence (−Dox) and presence of Hyaluronan (+HA) to stimulates CD44 expression. Cell lysates were collected and tested for the activity of MMP-2 (**a**), MMP-9 (**b**) at 0, 24 and 48 h upon CD44 up-regulation. Shown are the cropped blot images representing indicated proteins activity, and all the gels have been run under the same experimental conditions. The difference is considered statistically significant (student’s two-tailed t-test, ***P < 0.001)

Our results showing that increased expression of CD44 resulted in decreased expression of CD146 on the cell surface of BC cells, suggest that CD44 signaling may induce the release of CD146 from the cell surface. Recent studies have discussed the relationship between CD44 expression on cancer cell surface and activation of Matrix Metalloproteinases (MMPs) [27–30]. Based on these studies, we hypothesized that CD44-dependent release of CD146 may occur following an increase of MMPs activity. To test this hypothesis, we used casein zymography approach to evaluate MMPs activity in culture medium upon induction of CD44. Our results showed a 40% increase in the activity of MMP2 (Fig. 6a) 24 and 48 h post-CD44 induction, and 20% increase in the activity of MMP9 (Fig. 6b) after 24 h of CD44 induction.

### Determining the role of CD146 in breast cancer cell invasion

**Fig. 7:**
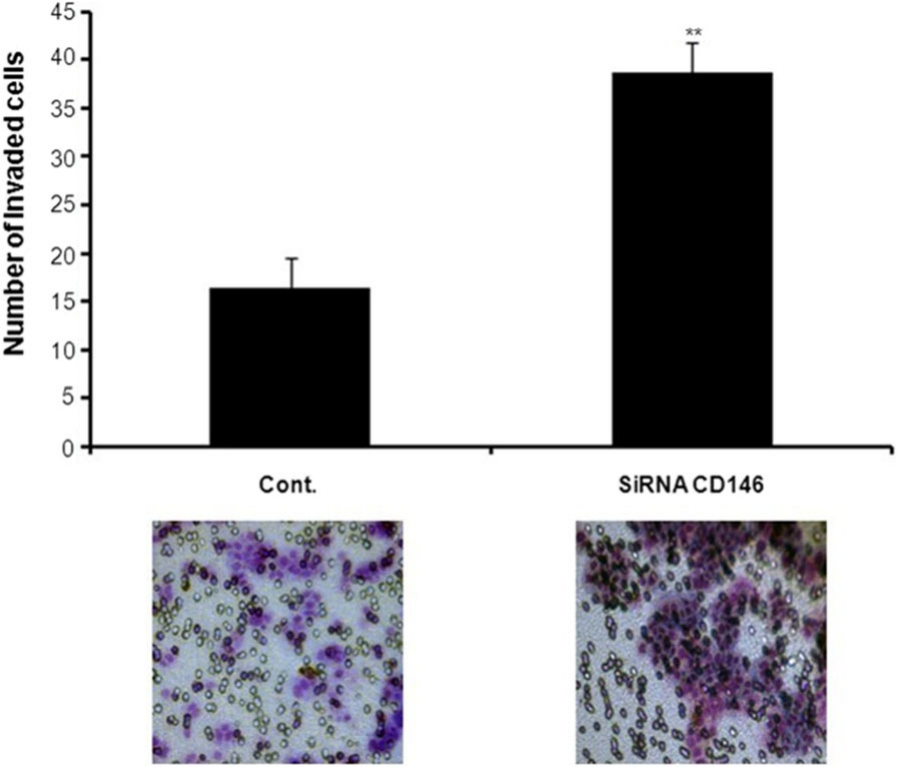
Functional validation of CD146 function in breast cancer cell invasion. Invasion of cells was demonstrated by Boyden chamber invasion assay. Note a marked induction in MCF7-B5-CD146 cell invasion capability after inhibiting CD146 expression using siRNA. Control cells were cultured in the same conditions (HA-, tet+) to induce CD146 and inhibit CD44, then it was transfected with scrambled siRNA as negative control for CD146 specific siRNA. All data have been done in triplicate and in three independent experiments. Representative images of Boyden chamber membranes represent the number of captured cells during the invasion, illustrating the difference in invaded cell numbers. The difference is considered statistically significant (student’s two-tailed t-test, **P < 0.001)

We have previously reported that CD44 induction enhances the invasiveness of BC cell lines in vitro, and induced breast tumor metastasis to the liver [3, 4]. This data prompted us to examine whether increased invasiveness may result from a decrease in CD146 expression. To test this hypothesis, we transiently transfected MCF7-B5-CD146 cells with a CD146-specific siRNA or a non-specific siRNA control oligonucleotide, and then determined the invasive ability of these cells using the Boyden chamber invasion assay. We observed greater than two-fold increase in the invasive capacity of the cells transfected with CD146-specific siRNA relative to the negative control (Fig. 7). This result suggests that loss of CD146 increased the invasive ability of MCF7-B5-CD146 cells and provides a link between our previous data, in which CD44 expression increased cellular invasiveness, and our present results, in which CD44 expression decreased the levels of cellular CD146.

## Discussion

The role of CD146 in promoting progression and metastasis of melanoma and PC has been widely documented [16, 31–33]. The upregulation of CD146 expression contributes to increased tumorigenicity, poor prognosis in metastatic melanoma [15, 34] and is believed to contribute to melanoma metastasis [35, 36]. Similarly, CD146 overexpression has been reported in metastatic PC cell lines but not in normal and non-metastatic cells [17, 18]. However, although the role of CD146 in these cancers was confirmed, the very little known about its role in BC metastasis is controversial [2, 20, 21, 23]. One group observed the expression of CD146 in normal breast tissue but only in 17% of the metastatic tumors, suggesting an inhibitory effect on BC metastasis [23]. In contrast, another group demonstrated high levels of CD146 expression in metastatic BC, restricted to the vascular endothelium, which is obvious because CD146 is known as a marker of endothelial cells [20].

Several lines of evidence suggest a link between CD44 signaling and CD146 expression. Our previous microarray data indicated that the upregulation and activation of CD44 resulted in the alteration of CD146 expression in MCF7-B5 cells (data not shown). Further, we have previously reported that the cytoskeletal actin filament associated protein, cortactin is a downstream target of CD44 signaling, a mechanism by which CD44 induces actin filament polymerization [3] and may potentiate BC motility and in-vivo cell invasion [37]. Further, CD146 is also associated with the cytoskeletal actin filament, suggesting that, like cortactin, CD146 may also contribute to CD44-dependent BC development [38, 39].

In the present study, we provide evidence to support our hypothesis that CD44 signaling contributes to the inhibition of cellular expression of CD146, which subsequently contributes to BC invasiveness. As matter of fact, clinical studies have reported a loss of CD146 in metastatic breast tumors supporting our current findings. First, we showed that the inducible expression and activation of CD44 resulted in a substantial decrease in the expression of cellular CD146 (Fig. 1) with an equally notable converse effect upon siRNA inhibition of CD44 expression (Fig. 2). Second, we observed an inverse relationship between CD44 and CD146 in late stage breast adenocarcinoma tissue samples relative to the adjacent normal tissue (Fig. 3; Table 1), thus supporting our in vitro results (Fig. 1). Furthermore, although there is a significant decrease in CD146 expression in late stage breast tumors, it is restricted to regions of neovascularization. Third, our immunofluorescence results confirm the Western blot data, demonstrating that although there was a significant increase in CD146 expression upon siRNA inhibition of CD44, there was alteration in the localization of CD146; It was absent from the surface of cells involved in making cell-cell contacts to being present on the surface of all cells (Fig. 4). Fourth, consistent with our immunofluorescence data, we observed that an increase in CD44 expression resulted in a decrease in *cellular* CD146 and an increase in *soluble* CD146 (Fig. 5). This result is further confirmed by the CD44-dependent activation of MMP (Fig. 6); these data suggest that MMP activation by CD44 resulted in the cleavage of CD146 from the surface of the cell. Finally, the decrease in CD146 subsequently promotes BC cell invasiveness (Fig. 7), suggesting that loss of cell surface CD146 is required for the promotion of BC invasiveness.

Two apparently contradicting reports studied the relevance of CD146 in BC cell motility and invasion. While, Zabouo et al., (2009) demonstrated that siRNA-dependent decrease in CD146 reduced the motility of BC cells; preliminary data from our group demonstrated that increase in CD146 expression significantly reduced cell invasion [2]. In this study, we confirmed the latter observation by providing substantial data demonstrating that the inhibition of CD146 expression significantly induced cell invasiveness. We have extended these observations and demonstrated an inverse relationship between the cell adhesion molecules, CD44 and CD146 in BC cell lines where there was an increase in CD44 expression resulting in the decrease in cellular expression of CD146. These data put together, suggest a model in which the expression and activation of CD44 results in the MMP-dependent cleavage of CD146 from the surface of the cell. The loss of CD146 from the cell surface further alters the intercellular interactions of cells within the microenvironment to subsequently induce the motility and invasiveness of BC cells, thereby resulting in tumor cell metastasis.

In this model, there are two main functions of CD146 within the tumor microenvironment. First, CD146 is greatly expressed by circulating endothelial and progenitor cells, which are responsible for the neovascularization process essential for tumor growth [9, 12], and as such plays a fundamental role in promoting angiogenesis and neovascularization [11]. Second, CD146 is known as an endothelial cell marker responsible for the tight adhesion between endothelial cells [40]. Therefore, it is conceivable that CD44-dependent expression of cell surface CD146 may have dual functions at different stages of BC tumor development that are most likely dependent on the tumor microenvironment. In early stages of BC, CD44 expression is low, resulting in higher levels of CD146 expression therefore promoting the tight adhesion between cells. During early stages of metastasis, the BC cell expression of CD44 is increased resulting in the elevation of MMP2 and MMP9 activation [41]. Moreover, CD44 acts as a docking site for MMP9, thus inducing its activation and redistribution to the cell surface, thereby bringing MMP9 to a direct proximity to cell surface, thus allowing MMP9 to maintain its proteolytic activity on the cell surface [30]. The proximity of MMPs to cell surface CAMs, such as CD146, would subsequently release proteins such as CD146 into the supernatant. The absence of CD146 on the cell surface would then decrease cell-cell adhesions, thereby promoting cell metastasis by degrading the tumor tissue basement membrane and extracellular matrix [30]. Soluble CD146 then acts as a chemotactic factor for the migration of circulating progenitor cells, which is a crucial step for neovascularization and pro-angiogenesis [41]. Once vascularization has been initiated, the upregulation of soluble CD146 intracellular levels enhances VEGFR2 expression and VEGF secretion, which in turn signals for angiogenesis [11].

## Conclusion

These results put together suggest that CD44 activates MMP resulting in the cleavage of CD146 from the cell surface, thereby promoting BC cell invasion. Here, we provide a possible mechanism by which CD146 suppresses BC progression as a target of CD44-downstream signaling, regulating neovascularization and cancer cell motility. CD146 may in fact exert two distinct functions in BC progression: i) the loss of cell surface CD146 promotes the motility and invasion of tumor cells; and ii) the released soluble form then initiates the neovascularization process at secondary sites of metastasis.
